# Articular fracture of the distal humerus classified Dubberley 2b: Case report of two patients and review of the literature

**DOI:** 10.1016/j.tcr.2024.101032

**Published:** 2024-04-10

**Authors:** Raphaël Fouché, Laela El Amiri, Nassim Bestandji, André-Pierre Uzel

**Affiliations:** a University of the French Antilles, Department of Orthopedic Surgery, University Hospital of Guadeloupe, Route de Chauvel, 97139 Les Abymes, Guadeloupe; b Department of Hand Surgery, SOS Main, CCOM, University Hospital of Strasbourg, FMTS, Icube CNRS 7357, University of Strasbourg, 10 Avenue Baumann, 67400 Illkirch, France

**Keywords:** Elbow, Humerus, Orthopedics, Humeral fractures, Intra-articular fractures

## Abstract

Articular fractures of the distal humerus are rare, and even rarer are fractures involving the trochlea and capitellum in a single fragment, with no associated comminution. These fractures are classified as 2a according to the Dubberley classification and are rarely described in the literature. Two cases of Dubberley 2a fractures were treated at our hospital. The first case, involving a 68-year-old patient, was treated with a medial and a lateral approach, combined with posteroanterior fixation using 3 Herbert screws. In the 2nd case, a 16-year-old male was treated with a single lateral approach, permitting fixation with two Herbert screws. One of the two screws is inserted into the bone at the edge of the cartilage, with an anteroposterior trajectory that leaves the cartilage intact. We opted mainly for posteroanterior screw fixation in subchondral bone, which is less damaging to articular cartilage and soft tissues and has already demonstrated its reliability. No associated lesions were found, and no complications were encountered. Results were excellent, with Mayo Elbow Performance Index (MEPI) scores of 95 and 100 respectively. Herbert screw fixation therefore appears to be an option of choice for these fractures, although comparative studies are needed to evaluate the different treatments available.

## Introduction

Frontal articular fractures of the distal end of the humerus are rare [1,2], and even rarer are fractures that involve the trochlea and capitellum in a single fragment. A classification was proposed in 2006 by Dubberley [3] to separate frontal articular fractures into 3 subtypes. Fractures involving a single fragment of the trochlea and capitellum without associated posterior comminution are then classified as 2a. There are a few series using Dubberley's [[3], [4], [5], [6], [7], [8], [9], [10], [11], [12]] classification; 2a fractures are found in 16.7 % of these series.

Theses fractures presents a flexion-extension elbow blockage. Radiological diagnosis using standard anteroposterior and lateral side views is often difficult. It is recommended to complete evaluation with a CT scan to assess joint damage precisely.

The treatment generally proposed is open reduction and internal fixation (ORIF), but osteosynthesis can be difficult due to the limited view and limited subchondral bone available for fixation.

We report two cases of Dubberley type 2a fracture.

## Cases report

### Case no 1

**Fig. 1:**
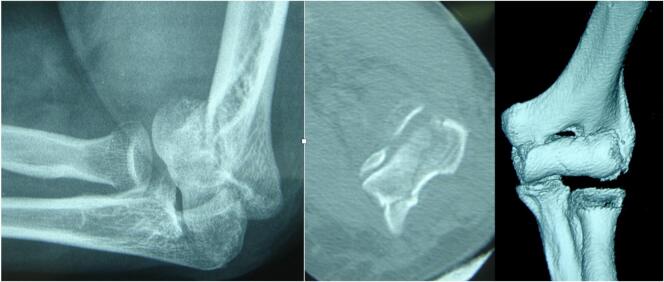
Initial radiographic evaluation of patient 1.

A 68-year-old woman, right-handed, was admitted on May 05, 2009 after falling on his bent elbow. The radiographs and a CT scan showed a single-fragment frontal fracture of the capitellum and trochlea, without posterior comminution, classified as type 2a in the Dubberley classification, (Fig. 1).

The patient underwent surgery 7 days after the trauma. With the patient in the supine position and a pneumatic tourniquet, the arm is placed in abduction. A double approach was used, the first lateral along the Kocher approach and a second posteromedial made through the space between the olecranon and the medial epicondyle, particular care is taken to visualize the ulnar nerve and avoid its injury. The double approach will allow easy reduction of the fragment through an anterior window, and the possibility of postero humeral extension for screw insertion on both sides. Osteosynthesis was performed with 3 posteroanterior Herbert screws. One screw is inserted medially and two laterally. In both approaches, particular care is taken to preserve the ligamentous planes that are essential for good elbow function. Reduction and fixation are facilitated using temporary K-wire. Intraoperative findings showed no cartilaginous impaction or free osteocartilaginous fragment. Joint stability testing revealed no ligament damage.

**Fig. 2:**
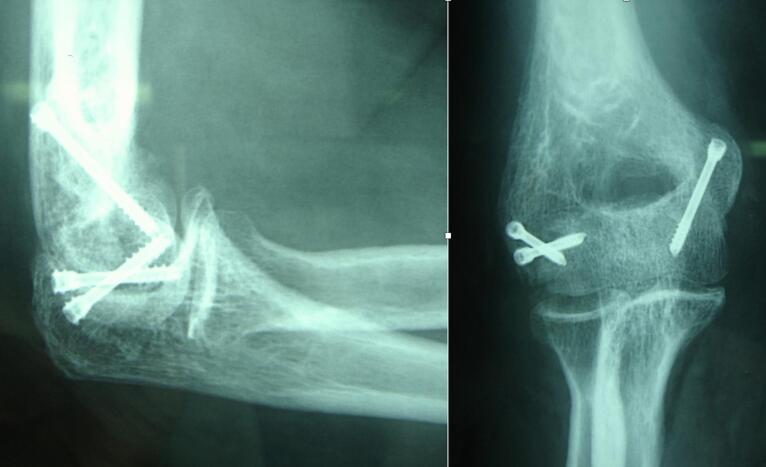
Elbow radiographs of patient 1 at 5-year follow-up.

The post-operative course was straightforward, and early rehabilitation in an articulated splint was started on day 10. Reduction was good, and consolidation was achieved by the second month, (Fig. 2).

**Fig. 3:**
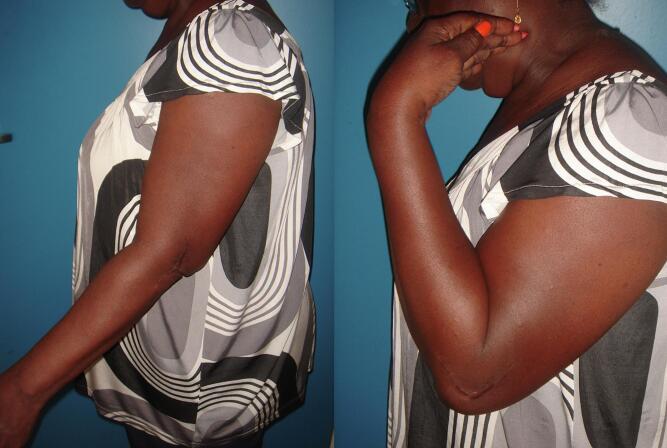
Patient 1 elbow range of motion at 5-year follow-up.

At 5 years, clinical evaluation based on the Mayo Elbow Performance Index (MEPI) [13] revealed an index of 95/100, which is excellent. She only reported pain sometimes during the evening or at bedtime. There was no axial deviation, and the elbow was stable. Mobility was 110/25/0 active and 120/25/0 passive, versus 140/0/5 on the healthy side. Pronosupination was not limited or painful, (Fig. 3). There was no evidence of epiphyseal osteonecrosis, and osteoarthritis.

### Case no 2

**Fig. 4:**
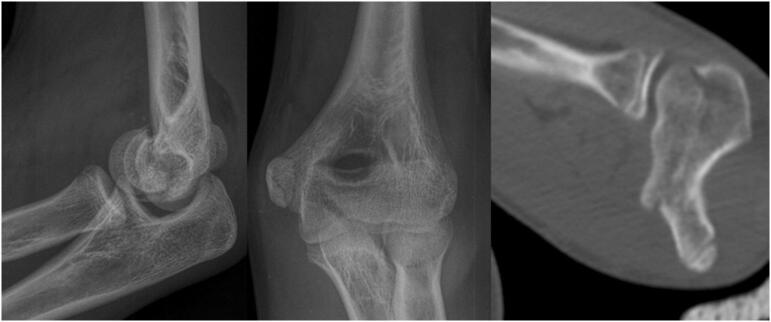
Initial radiographic evaluation of patient 2.

A 16-year-old man, right-handed, was admitted on March 11, 2020 after an injury that occurred during a basketball game, he fell on his left arm in extension. On initial radiological examination (standard X-rays and CT scan), the fracture was seen to involve the entire capitellum and most of the trochlea, but a small medial and distal corner of the trochlea remained unfractured, (Fig. 4). The lesion was then classified as Dubberley 2a.

**Fig. 5:**
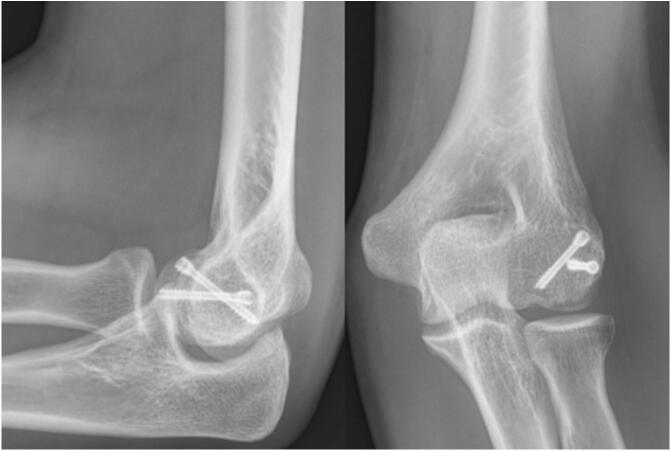
Elbow radiographs of patient 2 at 2-year follow-up.

The patient underwent surgery for ORIF the same day. The arm is placed in abduction. The same Kocher's lateral approach was used for reduction and fixation with 2 Herbert screws, the most medial screw is anteroposterior in the bone at the edge of the cartilage and the second is posteroanterior, (Fig. 5). A medial approach was not performed, as in Case 1, because CT scans showed almost no subchondral bone in the medial trochlea and the intraoperative findings revealed a cartilaginous flap medial to the trochlea, providing stability on this side. Intraoperative findings showed no cartilaginous impaction or free osteocartilaginous fragment. Testing revealed no ligament instability.

The immediate post-operative course was straightforward, and the patient was discharged from hospital the following day and early rehabilitation in an articulated splint was started on day 10. Bone consolidation was achieved at 2 months, and full range of motion was restored to the healthy side within 3 months (140/0/0 bilaterally).

**Fig. 6:**
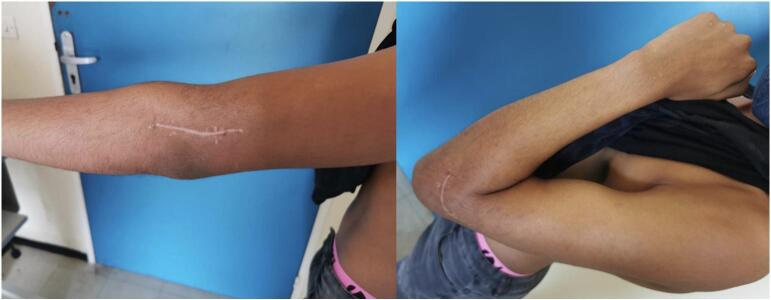
Patient 2 elbow range of motion at 2-year follow-up.

At 2 years, the elbow showed no signs of ligament instability, and range of motion were normal in both flexion-extension and pronosupination, (Fig. 6). Strength is normal and pain-free. The MEPI score is 100, which is excellent.

Radiologically, at 2 years, there was no evidence of osteonecrosis or osteoarthritis.

## Discussion

The outcome of the patients followed is excellent, with the pattern of fracture 2a, which is free of posterior and articular comminution, being a key explanatory factor. Indeed, Ashwood has shown that these are two criteria associated with poor results [6]. The effective fixation of the articular pavement and the integrity of the elbow ligaments enabled our patients to resume mobilization quickly, which is essential to prevent stiffening. It may be presumed that the good reduction achieved in both patients contributed to these good results.

Series on capitellar fractures generally show a high rate of associated lesions ranging from 24 to 60 % association [[3], [4], [5],^8^,^14^], the most common related injury is the fracture of the radial head. We also observe elbow dislocation, and injury to ligamentous structures, mainly the LCL.

In fractures classified as 2a, only 3 radial head fractures [5,10] and three LCL [7] lesions were identified in the series published in the literature. We didn't find nonunion in any series, and functional scores measured at follow-up were generally very good, with no MEPI score below 85.

The treatment most frequently proposed is osteosynthesis. Most often involving Herbert-type buried cannulated screw fixation, either trans-articular or posterior-anterior [[4], [5], [6], [7],^9^,^10^,^12^]. Other types of fixations include cancellous screws [3], bioabsorbable rods [6] and the Orthofix™ FFS system [8]. In a biomechanical study comparing 3 fixation methods for the capitellum, Herbert screws were found to provide better fixation than 4 mm cancellous screws [15]. Another cadaveric study [16] compares fixation of capitellum fractures with Herbert screws that are partially threaded versus Acutrac screws that are fully threaded, both screw designs are headless. Fully threaded screw provided better fixation stability with a significant difference against Herbert screw. The fully threaded design should therefore be recommended. Unfortunately, they are not available in our center, so we have used classic Herbert screws in our series. Posteroanterior fixation is discussed in Elkowitz's [15] article and shows better fixation in the subchondral bone than anteroposterior directed fixation. Another advantage of this type of fixation is that it preserves the soft tissues, compared with anteroposterior screw fixation which is harmful when the anterior tissues are retracted to drill and place the screw through the cartilage. In our technique, an anterior window in the approach is made for fragment reduction, while a posterior window is made for screw placement, thus avoiding protrusion into the olecranon fossa, a potential pitfall of anteroposterior screw fixation. At the same time, the anterior window allows us to monitor the length of the screws, which must not protrude into the articular cartilage.

Elbow arthroscopy [17] could be a source of innovation: the reduction, the fixation (percutaneous) and its approach would be less traumatic. However, existing series [[18], [19], [20]] do concern isolated fractures of the capitellum or trochlea. The more limited approach is an advantage for recovery in the follow-up phase but can cause real reduction difficulties when external manipulations are ineffective.

Total elbow arthroplasty could be studied as a solution in elderly patients with low functional demand, as it has already proved its worth against osteosynthesis of distal humerus articular fractures [21], mainly on comminuted fractures with osteoporosis, but in the context of a Dubberley 2a fracture, which is essentially a single-fragment fracture, it does not appear to be the treatment of choice. A study [22] reported a resurfacing of the radiocapitellar joint after an arthritic evolution of a Bryan and Morrey IV fracture, initially treated with Herbert screws. Another study [23] presents the case of a trochlea nonunion treated with an Elbow Hemiarthroplasty. Elbow arthroplasty offers interesting results [24] and may therefore be of interest in the complicated aftermath of coronal shear fractures.

Dubberley 2a fractures are a humeral fracture entity in their own right; they are rare and their treatment can be challenging. However, our series of 2 cases with screw fixation using Herbert screws shows good follow-up results.

## Funding

This research was performed as part of the employment of the authors, the hospital of Pointe à Pitre in Guadeloupe, France. The funder was not involved in the manuscript writing, editing, approval, or decision to publish.

## CRediT authorship contribution statement

**Raphaël Fouché:** Writing – original draft. **Laela El Amiri:** Writing – original draft. **Nassim Bestandji:** Writing – original draft. **André-Pierre Uzel:** Writing – review & editing.

## Declaration of competing interest

The authors report no conflicts of interest.
